# Efficacy of crizotinib and pemetrexed-based chemotherapy in Chinese NSCLC patients with *ROS1* rearrangement

**DOI:** 10.18632/oncotarget.12612

**Published:** 2016-10-12

**Authors:** Limin Zhang, Tao Jiang, Chao Zhao, Wei Li, Xuefei Li, Sha Zhao, Xiaozhen Liu, Yijun Jia, Hui Yang, Shengxiang Ren, Caicun Zhou

**Affiliations:** ^1^ Department of Medical Oncology, Shanghai Pulmonary Hospital and Thoracic Cancer Institute, Tongji University School of Medicine, Shanghai 200433, P.R. China; ^2^ Department of Lung Cancer and Immunology, Shanghai Pulmonary Hospital, Tongji University School of Medicine, Shanghai 200433, P.R. China

**Keywords:** non-small-cell lung cancer, ROS1 rearrangement, crizotinib, pemetrexed, thymidylate synthase

## Abstract

**Background:**

*ROS1* rearrangement is a novel molecular subgroup of non-small-cell lung cancer (NSCLC). This study aimed to investigate the efficacy of crizotinib and pemetrexed-based chemotherapy in Chinese NSCLC patients with *ROS1* rearrangement.

**Results:**

A total of 2309 patients received *ROS1* fusion detection and 51(2.2%) patients had *ROS1* rearrangement. There was no significant difference between ROS1 fusion-positive and fusion-negative cohorts in demographic data. For the *ROS1* fusion-positive patients, crizotinb-treated group had a higher overall response rate (ORR, 80.0%), disease control rate (DCR, 90.0%) and longer progression-free survival (PFS, 294 days) compared with the rates in pemetrexed-treated group (ORR, 40.8%; DCR, 71.4%; PFS, 179 days) and non-pemetrexed-treated group (ORR, 25.0%; DCR, 47.7%; PFS, 110 days). Besides, ORR, DCR and PFS were similar in three major ROS1 fusion partners. For the first-line treatment, patients received pemetrexed had a significant longer PFS than those received non-pemetrexed chemotherapy (209 vs. 146 days, *P* = 0.0107). In pemetrexed-treated cohorts, ROS1-positive patients with low TS expression had a statistically significant longer PFS than those with high TS expression (184 vs. 110 days, *P* = 0.0105).

**Materials and methods:**

We retrospectively identified patients with NSCLC who were screened for *ROS1* fusion using multiplex reverse transcription-polymerase chain reaction (RT-PCR) from October 2013 to February 2016. The thymidylate synthase (TS) mRNA levels were tested using quantitative real-time RT-PCR.

**Conclusions:**

Crizotinib was also highly active at treating Chinese NSCLC patients with *ROS1* rearrangement. TS expression could predict the efficacy of pemetrexed-based therapy in *ROS1* fusion-positive patients.

## INTRODUCTION

Lung cancer is the most common malignant tumor and the leading cause of cancer death worldwide, with non-small-cell lung cancer (NSCLC) patients accounting for 80–85% of its cases [1]. In the past 10 years, with the identification of oncogenic drivers, such as epidermal growth factor receptor (*EGFR*) mutation and anaplastic lymphoma kinase (*ALK*) rearrangements, the quality of life and prognosis of NSCLC patients with these driver mutations have been significantly improved when they were treated with small molecular tyrosine kinase inhibitors (TKIs) [2–4].

The c-ros oncogene 1 (*ROS1*) rearrangements, detected in 1%~2% of NSCLC [5–9] and up to 3% in lung adenocarcinoma [10, 11], represent a novel molecular subgroup of NSCLC. Similar to *ALK* fusions, *ROS1* rearrangement-positive patients had tendencies to be younger, never-smoker, and with adenocarcinoma histology [5–9]. *ROS1* and *ALK* are receptor tyrosine kinases (RTK), and both of them belong to the insulin receptor superfamily. The kinase domains of *ALK* and *ROS1* fusion proteins display highly homology, implying that *ALK*-TKI, such as crizotinib, will be also effective for *ROS1* rearrangement-positive NSCLC patients [12]. Preliminary data from a phase 1 clinical trial showed that crizotinib was highly active in *ROS1* fusion-positive patients [13]. Moreover, a retrospective study showed that *ROS1* fusion-positive NSCLC patients were greatly sensitive to crizotinib [14]. On the basis of the demonstration of substantial efficacy in the above phase I study, crizotinib has recently been approved by the United States Food and Drug Administration (FDA) as a treatment for patients with *ROS1* fusion-positive NSCLC. However, the studies above were carried out among Caucasian populations. It is still unknown about the efficacy of crizotinib in large-scale Chinese NSCLC patients with *ROS1* rearrangement.

Furthermore, at some point in the course of their disease, most patients with *ROS1* fusion-positive NSCLC will be treated with standard chemotherapeutic agents. Thus, establishing the efficacy of chemotherapeutic agents in this genetically defined subset of patients is clinically relevant. It has been previously shown that *ROS1* fusion-positive patients are responsive to pemetrexed-based chemotherapy [15, 16]. Kim et al. reported that 5 NSCLC patients with *ROS1* rearrangement had a better responsive to pemetrexed than thWose without *ROS1*/*ALK* rearrangement [15]. Another retrospective study showed that *ROS1* fusion-positive patients who received pemetrexed-based regimens had a better ORR, DCR and longer PFS compared with patients harboring other driver mutations [16]. These findings have led to the notion that *ROS1* rearrangement may serve as a predictive biomarker of enhanced pemetrexed sensitivity. In addition, many prior studies indicated that TS levels can predict the response of pemetrexed-based chemotherapy in NSCLC [17–22]. However, whether TS expression levels are correlated with the response of pemetrexed-based chemotherapy in *ROS1* rearrangement NSCLC patients remains controversial.

Herein, we analyzed arguably the largest cohorts to assess the efficacy of crizotinib and pemetrexed-based regimen in Chinese NSCLC patients with *ROS1* rearrangement in this study. Meanwhile, we also investigated whether TS mRNA levels were associated with the response of pemetrexed-based regimen in *ROS1* rearrangement NSCLC patients.

## RESULTS

### Patients' characteristics

**Figure 1:**
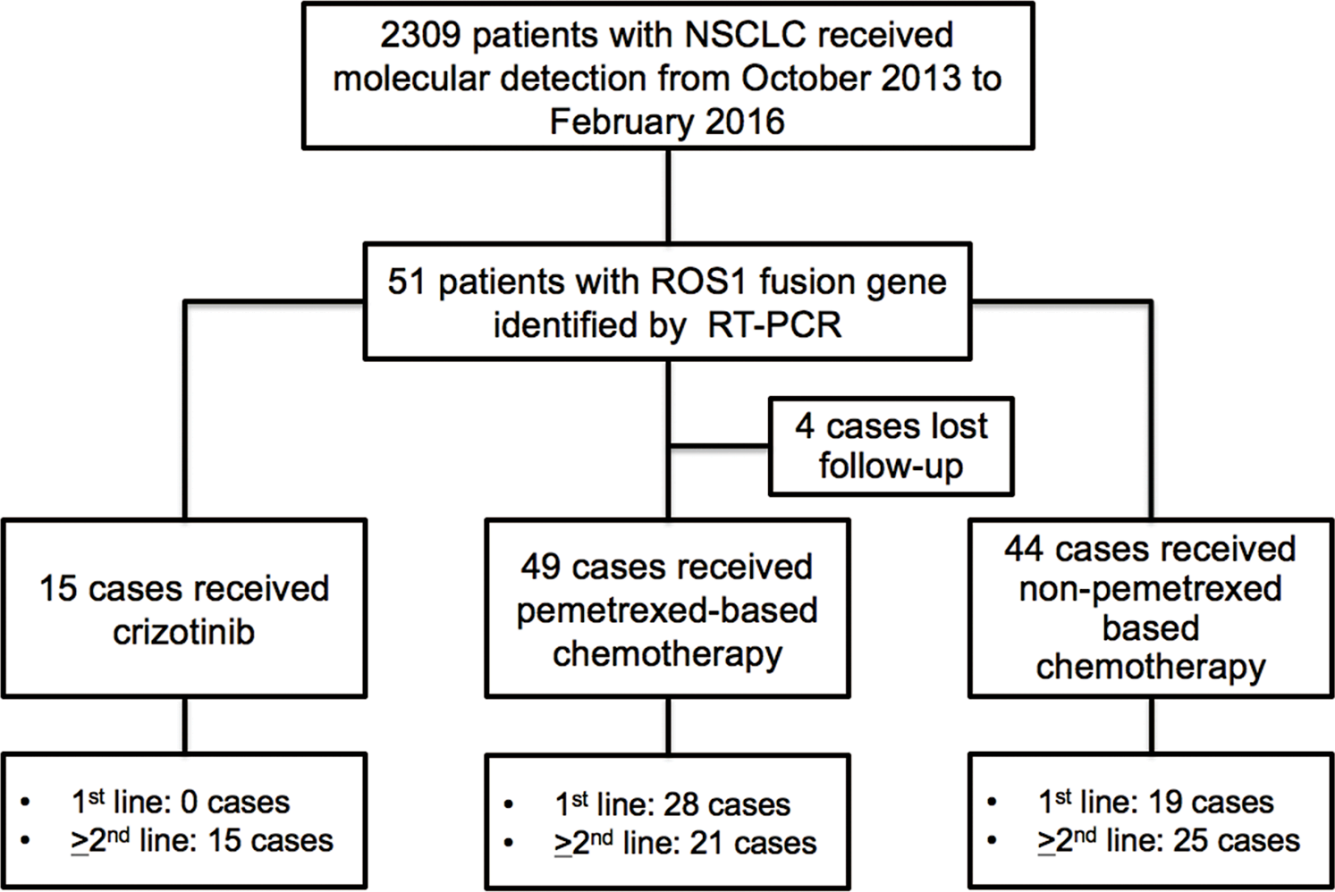
Flow chart of the study design

2309 patients with NSCLC received *ROS1* rearrangement detection from October 2013 to February 2016 were included in this study. 51 patients (2.2%) were identified as *ROS1* rearrangement-positive. Among those, 15 patients received crizotinib (1st line treatment, *n* = 0; and ≥ 2nd line treatment, *n* = 15), 49 patients received pemetrexed-based chemotherapy (1st line treatment, *n* = 28; and ≥ 2nd line treatment, *n* = 21), 44 patients received non-pemetrexed-based chemotherapy (1st line treatment, *n* = 19; and ≥ 2nd line treatment, *n* = 25), and 4 cases lost follow-up (Figure 1). Their baseline clinical characteristics are outlined in Table 1. No statistical significance was observed based on age (*P* = 0.248), sex (*P* = 0.146), smoking history (*P* = 0.882), pathology type (*P* = 0.961), and ECOG performance status (*P* = 0.431) between *ROS1* fusion-positive and *ROS1* fusion-negative patients.

**Table 1:** Clinical characteristics of all included patients at baseline

Characteristic	Total (*n* = 2309)		ROS1 negative (*n* = 2262)		ROS1 positive (*n* = 47)		*P* value
	*n*	%	*n*	%	*n*	%	
**Age (years)**							
Median	61		64		57		
Range	27–82		27–82		31–77		
< 65	1486	64.4%	1452	64.2%	34	72.3%	0.248
≥ 65	823	35.6%	810	35.8%	13	27.7%	
**Sex**							
Male	894	38.7%	871	38.5%	23	48.9%	0.146
Female	1415	61.3%	1391	61.5%	24	51.1%	
**Smoking History**							
Never-smoker	1648	71.4%	1614	71.4%	34	72.3%	0.882
Former/current smoker	661	28.6%	648	28.6%	13	27.7%	
**Pathology Type**							
Adenocarcinoma	1811	78.4%	1774	78.4%	37	78.7%	0.961
Non-adenocarcinoma	498	21.6%	488	21.6%	10	21.3%	
**ECOG Performance Status**							
0–1	2055	89.0%	2011	88.9%	44	93.6%	0.431
≥ 2	254	11.0%	251	11.1%	3	6.4%	

EGFR, epidermal growth factor receptorr; ECOG, Eastern Cooperative Oncology Group.

### Effect of crizotinib treatment in NSCLC patients with *ROS1* fusion partners

**Figure 2:**
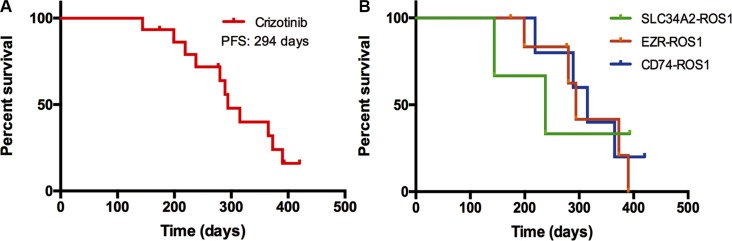
(**A**) Progression-free survival (PFS) of *ROS1* fusion-positive patients treated with crizotinib. (**B**) Comparison of PFS in three major *ROS1* fusion patterns-positive patients treated with crizotinib.

47 *ROS1* fusion-positive samples were reconfirmed by means of direct sequencing. The most common *ROS1* fusion partner was *CD74*, accounting for 40.4% (19 of 47); other partner genes included *EZR* in 13 (27.7%) patients, *SLC34A2* in 8 (17.0%), *SDC4* in 6 (12.8%), and *GOPC* in 1 (2.1%); the sequence of the *ROS1* fusion-positive patients in our study were shown in Supplementary Figure S1. According to the frequency of *ROS1* fusion partners, we classified these patients into 4 subgroups, patients with *CD74-ROS1*, *SLC34A2-ROS1*, *EZR-ROS1*, and other partner genes (including *SDC4-ROS1*, *GOPC-ROS1*). The clinical characteristics of the four subgroups are listed in Table 2. The tumor response was evaluated in 15 patients who received oral crizotinib with advanced NSCLC and *ROS1* fusion-positive. Among them, five had *CD74-ROS1* fusion, three had *SLC34A2-ROS1* fusion, and seven had *EZR-ROS1* fusion. For crizotinib treatment, one patient had a complete response, 11 had a partial response and three attained stable disease. However, there was no distinct correlation between the *ROS1* fusion partners and the tumor response of crizotinib treatment (Supplementary Figure S2). The median PFS time was 294 days (Figure 2A), and no statistical significance was observed in PFS among the three different *ROS1* fusion partners (Figure 2B).

**Table 2:** Clinical characteristics and comparison among 4 ROS1 fusion partners

Characteristics	CD74-ROS1	SLC34A2-ROS1	EZR-ROS1	Others
**Patients**	**19 (40.4%)**	**8 (17.0%)**	**13 (27.7%)**	**7 (14.9%)**
**Age (years)**				
Median	19 (38–73)	8 (35–72)	13 (31–77)	7 (44–76)
< 65	15 (78.9%)	4 (50.0%)	11 (84.6%)	4 (57.1%)
≥ 65	4 (21.1%)	4 (50.0%)	2 (15.4%)	3 (42.9%)
**Sex**				
Male	5 (26.3%)	8 (100%)	6 (46.2%)	4 (57.1%)
Female	14 (73.7%)	0 (0.0%)	7 (53.8%)	3 (42.9%)
**Smoking History**				
Never-smoker	16 (84.2%)	4 (50.0%)	11 (84.6%)	3 (42.9%)
Former/current smoker	3 (15.8%)	4 (50.0%)	2 (15.4%)	4 (57.1%)
**Pathological Type**				
Adenocarcinoma	15 (78.9%)	6 (75.0%)	11 (84.6%)	5 (71.4%)
Non-adenocarcinoma	4 (21.1%)	2 (25.0%)	2 (15.4%)	2 (28.6%)
**ECOG Performance Status**				
0–1	19 (100%)	8 (100%)	12 (92.3%)	5 (71.4%)
2–3	0 (0.0%)	0 (0.0%)	1 (7.7%)	2 (28.6%)

Others included SDC4-ROS1 & GOPC-ROS1.

### Treatment response in different therapeutic group

**Figure 3:**
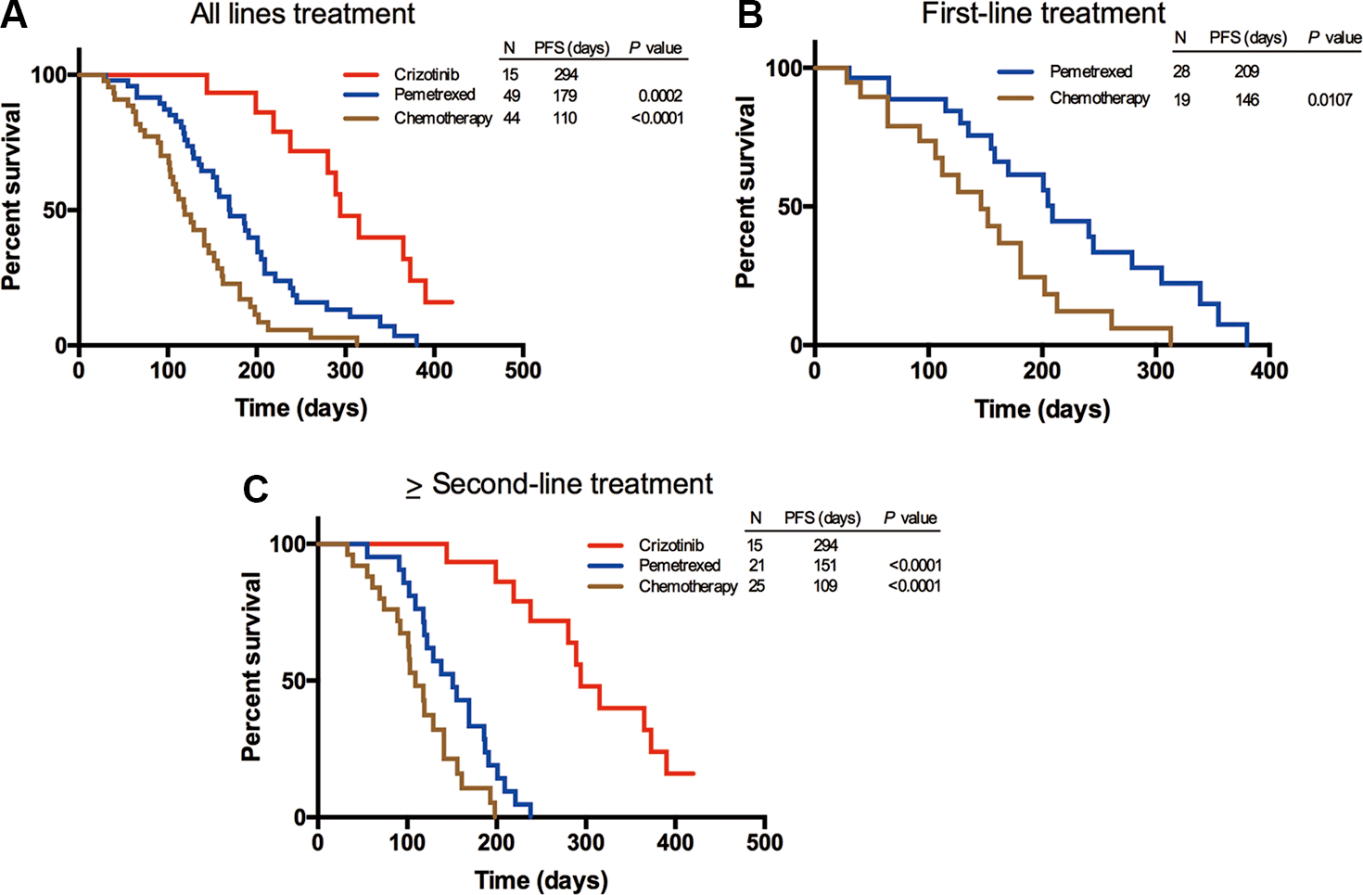
Progression-free survival (PFS) of *ROS1* fusion-positive patients treated with cizotinib, pemetrexed-based chemotherapy and non-pemetrexed-based chemotherapy, respectively (**A**) Comparison of PFS in *ROS1* fusion-positive patients who received cizotinib, pemetrexed-based chemotherapy or non-pemetrexed-based chemotherapy as their any line treatment. (**B**) Comparison of PFS in *ROS1* fusion-positive patients who received pemetrexed-based chemotherapy or non-pemetrexed-based chemotherapy as their first line treatment. (**C**) Comparison of PFS in *ROS1* fusion-positive patients who received cizotinib, pemetrexed-based chemotherapy or non-pemetrexed-based chemotherapy as their ≥ second-line treatment.

In the light of different therapeutic regimens that *ROS1* fusion-positive patients received, we divided them into three groups: crizotinib-treated group, pemetrexed-treated group and non-pemetrexed-treated group. Among patients receiving cizotinib, pemetrexed-based chemotherapy and non-pemetrexed chemotherapy in any line treatment, both crizotinib-treated and pemetrexed-treated groups showed a longer PFS compared with the non-pemetrexed-treated group, with the mPFS of 294, 179, and 110 days, respectively (Figure 3A). There was a statistically significant difference in PFS among the crizotinib-treated group, pemetrexed-treated group (*P* = 0.0002) and non-pemetrexed-treated group (*P* < 0.0001). In the above-mentioned subgroups of patients who received pemetrexed-based and non-pemetrexed-treated as the first-line treatment, the difference in PFS was observed between the pemetrexed-treated group and non-pemetrexed-treated group (209 vs. 146 days, *P* = 0.0107) (Figure 3B). Similarly, among these patients who received three therapeutic regimens as the ≥ second-line treatment, a statistically significant difference was also observed in PFS among the crizotinib-treated group (294 days), pemetrexed-treated group (151 days, *P* < 0.0001) and non-pemetrexed-treated group (109 days, *P* < 0.0001) (Figure 3C). Taken together, these results showed that the efficacy of either crizotinib or pemetrexed-based chemotherapy was better than that of non-pemetrexed chemotherapy regimens in the *ROS1* fusion-positive patients.

The tumor response of patients who received pemetrexed-based or non-pemetrexed-based chemotherapy was shown in Table 3. Patients received chemotherapy as a first-line treatment, in pemetrexed-treated group, none patient had CR, 15 achieved PR, eight had SD, five had PD; in non-pemetrexed-treated group, none patient had CR, seven achieved PR, five had SD, seven had PD. The *ROS1* fusion-positive NSCLC patients who received pemetrexed-based chemotherapy had a relatively better ORR or DCR than those treated with non-pemetrexed-based chemotherapy; however, the difference did not reach the statistical significance (ORR, 53.6% vs. 36.8%, *P* = 0.259; DCR, 82.1% vs. 63.2%, *P* = 0.143). Patients received chemotherapy as a ≥ second-line treatment, in pemetrexed-treated group, none patient had CR, five achieved PR, seven had SD, seven had PD, and two had NE; in non-pemetrexed-treated group, none patient had CR, four achieved PR, five had SD, 13 had PD, and three had NE. The *ROS1* fusion-positive NSCLC patients who received pemetrexed-based chemotherapy also had a relatively better ORR or DCR than those treated with non-pemetrexed-based chemotherapy; however, it did not have the statistically difference (ORR, 23.8% vs. 16.0%, *P* = 0.770; DCR, 57.1% vs. 36.0%, *P* = 0.152).

**Table 3:** Tumor response in patients received chemotherapy according to RECIST

		Pemetrexed-based	Non-pemetrexed based	*P* value
First-line treatment		*n* = 28	*n* = 19	
	CR	0	0	
	PR	15	7	
	SD	8	5	
	PD	5	7	
	NE	0	0	
	ORR	15 (53.6%)	7 (36.8%)	0.259
	DCR	23 (82.1%)	12 (63.2%)	0.143
≥ Second-line treatment		*n* = 21	*n* = 25	
	CR	0	0	
	PR	5	4	
	SD	7	5	
	PD	7	13	
	NE	2	3	
	ORR	5 (23.8%)	4 (16.0%)	0.770
	DCR	12 (57.1%)	9 (36.0%)	0.152

CR, complete response; PR, partial response; SD, stable disease; PD: progression disease; NE, Not evaluable; ORR: overall response rate; DCR: disease control response.

### Correlation of TS RNA levels with PFS on pemetrexed-based chemotherapy in *ROS1*-positive NSCLC

**Figure 4:**
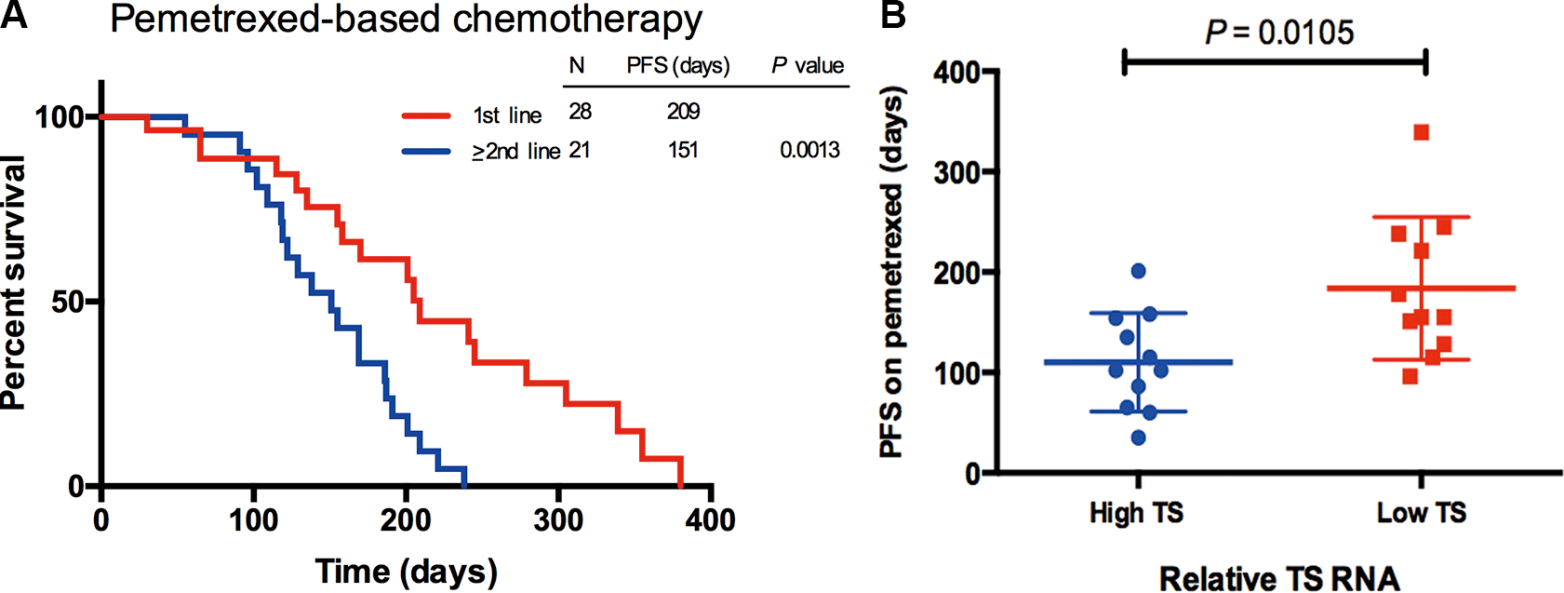
(**A**) Comparison of progression-free survival (PFS) in *ROS1* fusion-positive patients who received pemetrexed-based chemotherapy as 1st line or > 2nd line treatment. (**B**) Correlation of tumor thymidylate synthase (TS) RNA levels with PFS of *ROS1* fusion-positive patients treated with pemetrexed-based chemotherapy. TS RNA levels were compared with the median TS value of control cases of NSCLC.

In the pemetrexed-treated group, a statistically significant difference was observed in PFS between the 1st line and ≥ 2nd line treatment (209 vs.151 days, *P* = 0.0013) (Figure 4A). In order to uncover the relation of TS levels and the efficacy of pemetrexed, we detected the TS mRNA levels in the pemetrexed-treated group of *ROS1* fusion-positive patients. 22 of 49 *ROS1* fusion-positive patients had sufficient tumor specimen for the RT-PCR, including 11 patients with low TS expression and 11 patients with high TS expression. From Figure 4B, the PFS of the *ROS1* fusion-positive patients with low TS expression was statistically significant longer than those with high TS expression (184 vs. 110 days, *P* = 0.0105). This result suggested that TS levels can determine different response of *ROS1*-positive patients to pemetrexed.

## DISCUSSION

This is the first large-scale retrospective study to comprehensively investigate the efficacy of crizotinib, pemetrexed-based and non-pemetrexed-based chemotherapy in Chinese NSCLC patients with *ROS1* fusion-positive. We discovered that there was no apparent correlation between the PFS of patients treated with crizotinib and the *ROS1* fusion partners. The current study indicated that both crizotinib-treated and pemetrexed-treated groups had a significant longer PFS than non-pemetrexed-treated group, regardless of the lines of treatment. Besides that, in pemetrexed-treated group, a statistically significant difference was observed in PFS between the 1st and > 2nd regimens. Moreover, we found that *ROS1* fusion-positive patients treated with pemetrexed-based chemotherapy with low TS expression had an obvious longer PFS than those with high TS expression.

The frequency of *ROS1* rearrangement was 2.2% among unselected NSCLC patients in our study, which was a little bit higher than the records in previous studies [5–9]. In accordance with the previous study of Cai et al. [9], our results showed that *ROS1* rearrangement was not prone to be younger, never-smoker, and with adenocarcinoma histology on the basis of the subgroup analysis; however, all of which were opposed to the other previous studies [5–8]. The discrepancy might be ascribed to that some patients in the present study were selected from wild-type *EGFR* and wide-type *ALK* population. Therefore, we found the incidence of *ROS1* rearrangement was relatively higher than that in other studies [10, 11].

Several clinical data, including two Chinese clinical reports, have demonstrated the efficacy of crizotinib in NSCLC patients with *ROS1* rearrangement [23–28]. Our results were compatible with the European retrospective study of Julien Mazières et al., who reported that *ROS1* fusion patients treated with crizotinib showed a median PFS of 9.1 months [14], and the phase II study in 2016 ASCO, which reported that *ROS1* fusion patients treated with crizotinib showed the ORR of 69.3% and a median PFS of 13.4 months. We also found that patients with diverse *ROS1* fusions did not display significantly different efficacy, and no apparent correlation was detected between the specific *ROS1* fusion partner and PFS of *ROS1* fusion-positive patients treated with crizotinib in China, both of which were in line with a recently published prospective phase I study of crizotinib [13]. However, we should need large *ROS1* fusion-positive NSCLC patients treated with crizotinib, cell line models and experimental animal models to further demonstrate these results. Moreover, our results further verify that the efficacy of crizotinib is definitive among Chinese *ROS1* fusion-positive NSCLC patients.

Consistent with *ALK*-fusion NSCLC patients and with the previous studies, we found pemetrexed-based chemotherapy showed a better effect in *ROS1* fusion-positive patients than non-pemetrexed-based chemotherapy [14–16, 29, 30]. We found that the PFS of patients who treated with pemetrexed-based chemotherapy was 179 days, which was shorter than that in a European prior retrospective study (7.2 months) [14], or in a recently published retrospective study (7.5 months) which was carried out in Taiwan [16]. The difference can be triggered by the study population, tumor heterogeneity and previous treatments of patients. Intriguingly, we observed pemetrexed-based chemotherapy can be preferentially used as the 1st line treatment in *ROS1* fusion-positive NSCLC patients when they cannot receive crizotinib treatment.

As is known, TS is an important folate enzyme which can be mainly targeted by pemetrexed. Studies have demonstrated that the TS expression was associated with the treatment efficacy of pemetrexed in NSCLC patients, with low of TS expression conferring increased sensitivity to pemetrexed [17–22, 31]. Although a retrospective study suggested that H-scores of TS protein levels of *ROS1* fusion-positive patients were not associated with the PFS of pemetrexed therapy [16], the immunohistochemical staining which was adopted to assess TS levels is less sensitive and semiquantitative than quantitative real-time RT-PCR. A host of studies manifested that TS RNA level can predict the sensitivity of pemetrexed in NSCLC patients [18, 19, 22, 31]. In addition, Alice Shaw et al. reported that TS RNA level was inversely associated with the efficacy of pemetrexed in *ALK* fusion-positive NSCLC [31]. Kim et al. discovered that the *ROS1* fusion-positive HCC78 cell line had low TS expression and the most sensitivity to pemetrexed in comparison to other NSCLC cell lines [15]. By using quantitative real-time RT-PCR to measure the mRNA levels of TS, we discovered that *ROS1*-positive patients who had lower level of TS RNA showed better susceptibility to pemetrexed-based chemotherapy. Randomized clinical studies should be carried out to demonstrate these results.

Several limitations cannot be avoided completely in this study. First, the number of patients who had particular *ROS1* fusion partner treated with crizotinib was relatively small. Second, NSCLC patients enrolled in our study were drawn from the single-institution series. However, considering the relatively larger sample size, the current data can lay the foundation to further large-scale prospective studies of pemetrexed-based chemotherapy in Chinese *ROS1* fusion-positive patients. Third, this study was a retrospective study, which might have induced selection bias. Therefore, the findings in this study need to be validated in prospective trials with large scale.

In conclusion, our study suggests that crizotinib and pemetrexed-based chemotherapy are effective to patients with *ROS1* rearrangement-positive, and the relative expression of TS RNA can predict the sensitivity of *ROS1*-positive patients to pemetrexed.

## MATERIALS AND METHODS

### Study population and data collection

This study was carried out in a group of NSCLC patients received *ROS1* rearrangement detection by reverse transcriptase polymerase chain reaction (RT-PCR) assay between October 2013 and February 2016 at Shanghai Pulmonary Hospital, Tongji University School of Medicine, Shanghai, China. Pathological diagnosis and staging was carried out according to the staging system of the 2009 International Association for the Study of Lung Cancer (version 7). As for the paraffin-embedded and formalin-fixed samples, all samples were reviewed by pathologists to confirm the tumor histology and the tumor cells over 30% of the samples. As for the cytological samples, once the operator acquired the tumor biopsy tissue, it was separated into two parts: one part was for pathologic analysis, and another part, which was preserved for extracting mRNA, was directly put in an RNase-free Eppendorf tube containing 500 μl of RNAlater (Cat No.AM 7021, life technologies) and stored at −80°C until the analyses were performed.

Patients' medical records were reviewed to evaluating the clinicopathological features and treatment regimens. All the eligible patients' clinical data included the age, gender, smoking status, histological type, Eastern Cooperative Oncology Group (ECOG) performance status (PS) and previous treatment regimens. Nonsmokers were defined as patients with the smoking dose of < 100 cigarettes in their lifetime. Clinical responses were evaluated according to the response evaluation criteria in solid tumors (RECIST) version 1.1. PFS was measured from the first day of treatment until either tumor progression or death. This study was approved by Shanghai Pulmonary Hospital Ethics Committee. Each patient had signed a written informed consent before the study started.

### RNA preparation and reverse transcription

Total RNA was extracted from tissue samples using either RNeasy Mini Kit (Qiagen, Hilden, Germany) or AmoyDx RNA Kit (Amoy Diagnostics Co, Xiamen, China). The quantity and quality of extracted RNA was measured by NanoDrop 2000 Spectrophotometer (Thermo Scientific, Waltham, USA). Then, the extracted RNA was reversetranscribed to complementary DNA (cDNA) at 42°C for 1 hour, followed by 95°C for 5 min.

### *ROS1* rearrangements detection

*ROS1* rearrangements were identified by an AmoyDx^®^ *ROS1* fusion gene detection kit (Amoy Diagnostics Co., Ltd, Xiamen, China). The patterns of *ROS1* rearrangements were detected in our study as previously described [9]. The RT-PCR conditions of cDNA was as follows: one cycle of 95°C for 5 min; followed by 15 cycles of denaturation at 95°C for 25 s, annealing at 64°C for 20 s and elongation at 72°C for 20 s to ensure the specificity; and up to 31 cycles of 93°C for 25 s, 60°C for 35 s (data collection) and 72°C for 20 s. β-actin was used as an internal reference gene to ensure the quality of the extracted RNA and *ROS1*-rearranged DNA was used as positive control.

### Measurement of TS RNA levels in tumor tissues

The TS RNA level was measured by quantitative RT-PCR methodology using SYBR Premix Ex Taq (TaKaRa) and an MX3000P instrument. The sequences of primers for TS and Glyceraldehyde 3-phosphate dehydrogenase (GAPDH) reference gene were as follows:

TS forward 5′-GGCCTCGGTGTGCCTTT-3′, TS reverse 5′-GATGTGCGCAATCATGTACGT-3′; GAPDH forward 5′-AGGGCTGCTTTTAACTCTGGT-3′;GAPDH reverse5′-CCCCACTTGATTTTGGAGGG A-3′; Control cases of *ROS1* fusion-negative, EGFR and KRAS wild type NSCLC were also assessed to establish a median TS mRNA level.

### Statistical analysis

Categorical variables were compared using χ2 test or Fisher exact test when necessary. PFS was estimated by the Kaplan-Meier method, and the log-rank test was used to compare the difference between the groups. A Cox regression model was used to calculate hazard ratio (HR) and its 95% confidence interval (CI). Statistical analysis was performed using SPSS version 22.0 software (IBM, Armonk, NY). All *P* values were two-sided, and a *P value* of < 0.05 was considered statistically significant.

## SUPPLEMENTARY MATERIALS FIGURES


