# Supplementary material for: Efficacy of crizotinib and pemetrexed-based chemotherapy in Chinese NSCLC patients with ROS1 rearrangement
Source: Oncotarget. 2016 Oct 12;7(46):75145–54. doi: 10.18632/oncotarget.12612 (PMC5342729; doi:10.18632/oncotarget.12612)
Supplement: Supplementary file 1 [file oncotarget-07-75145-s001.pdf]

Efficacy of crizotinib and pemetrexed-based chemotherapy in Chinese NSCLC patients with *ROS1* rearrangement

Supplementary Materials

A **ROS1 (exon32); SLC34A2 (exon14 del):**

ROS1 exon32-TTTTACTCCCTTCTAGTAATTTGGGAATGCCTGGTTTATTTGGGACTCCAG

CCTGAGCCTCTCTGCTAATGGTTATGTTATCAAAGGTCTCAGGAGCC-SLC34A2 exon14

del

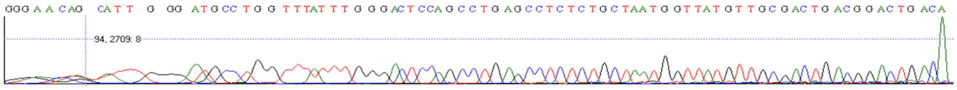

B **ROS1 (exon32) ;CD74 (exon6):**

ROS1 exon32-TTTTACTCCCTTCTAGTAATTTGGGAATGCCTGGTTTATTTGGGACTCCAG

CTTTCGGTGGAGCGTCAGTGGGCTTTTGCTCCAAGGAGTGCCTGCTCATTTC-CD74

exon6

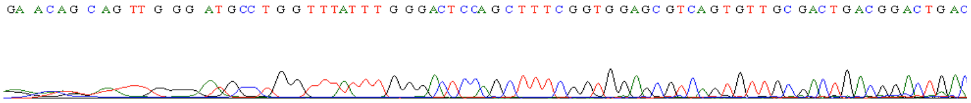

C **ROS1 (exon32) ;SDC4 (exon2):**

ROS1 exon32-TTTTACTCCCTTCTAGTAATTTGGGAATGCCTGGTTTATTTGGGACTCCAG

CCAGATCTCCAGAGCCAGACAGCTCAAAGTCATCAGATTCCTGCCCGGGCC-SDC4

exon2

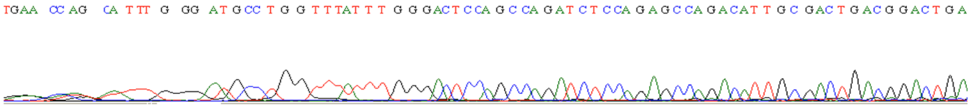

D **ROS1 (exon32); SDC4 (exon4):**

ROS1 exon32-TTTTACTCCCTTCTAGTAATTTGGGAATGCCTGGTTTATTTGGGACTCCAG

CTGCCAGGACCTCCGTTCTCTCAAAGATGTTGCTGCCCTGCACAGTGCTGG-SDC4

exon4

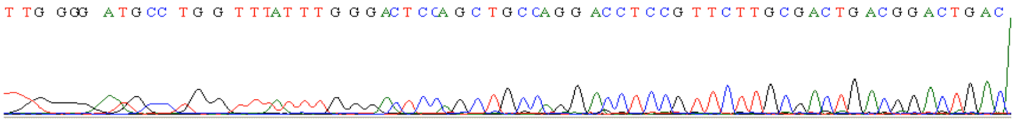

E ROS1 (exon34) ;SLC34A2 (exon4):

ROS1

exon34-TATTCCAACTATAATAGTAAGTATGAAACTTGTTTCTGGTATCCAAAAATCAT

CTCCAACCAGCTGGAAGGCGCTACTAAGAATATCCAGGGAGCACACGAA-SLC34A2

exon4

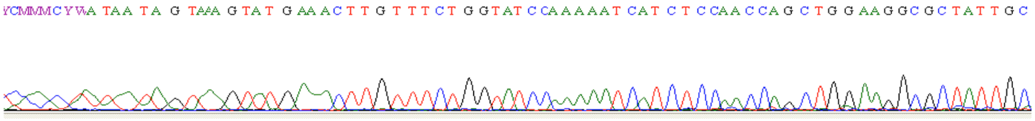

F ROS1 (exon34); SLC34A2 (exon14 del):

ROS1

exon34-TATTCCAACTATAATAGTAAGTATGAAACTTGTTTCTGGTATCCAAAAATCAT

CCTGAGCCTCTCTGCTAATGGTTATGTTATCAAAGGTCTCAGGAGCC-SLC34A2 exon14

del

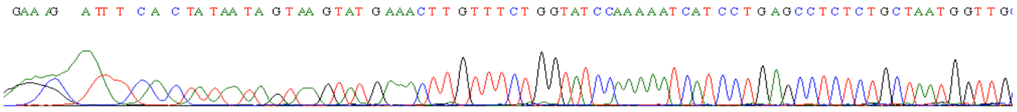

G ROS1 (exon34); CD74 (exon6):

ROS1

exon34-TATTCCAACTATAATAGTAAGTATGAAACTTGTTTCTGGTATCCAAAAATCAT

CTTTCGGTGGAGCGTCAGTGGGCTTTTGCTCCAAGGAGTGCCTGCTCATTTC-CD74

exon6

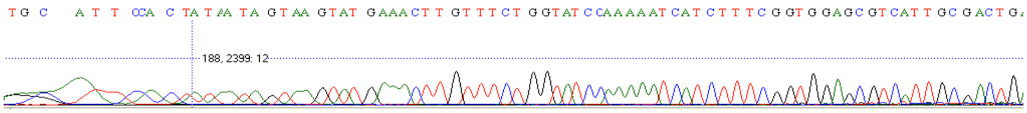

H ROS1 (exon34); EZR (exon10):

ROS1

exon34-TATTCCAACTATAATAGTAAGTATGAAACTTGTTTCTGGTATCCAAAAATCAT

CTCTCTCTGCCTTCTTTGTCTTCTCCTCATAGTCCTGCAGCCGCAGCATCAAC-EZR

exon10

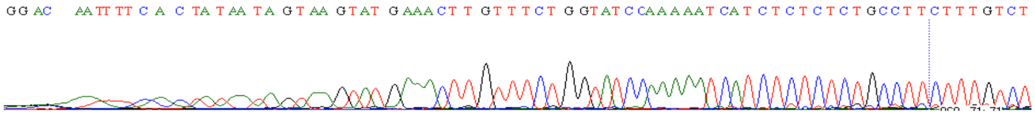

I ROS1 (exon35); GOPC (exon8):

ROS1

exon35-CACTGTCACCCCTTCCTTGGCACTTTTTTGATTCTTTAATCTTCTATGCCAGA

CTTGTAATACTTTGATTTCCCCACTTGTGTCTTTGCAACTAGCACCAGGGTTA-GOPC

exon8

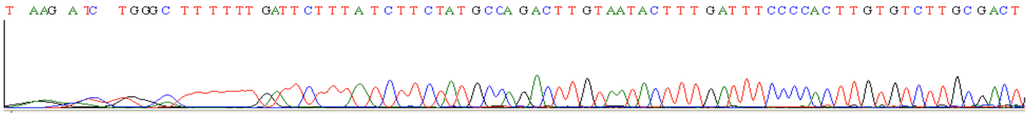

J ROS1 (exon32); SLC34A2 (exon4):

ROS1 exon32-TTTTACTCCCTTCTAGTAATTTGGGAATGCCTGGTTTATTTGGGACTCCAG

CTCCAACCAGCTGGAAGGCGCTACTAAGAATATCCAGGGAGCACACGAA-SLC34A2

exon4

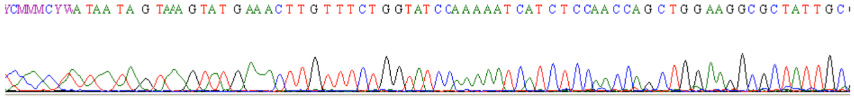

Supplementary Figure S1: Sequences of the different ROS1 fusion variants identified by a multiplex RT-PCR were reconfirmed by direct sequencing. (**A**) ROS1 (exon32); SLC34A2 (exon14 del) in four cases, (**B**) ROS1 (exon32) ;CD74 (exon6) in two cases, (**C**) ROS1 (exon32) ;SDC4 (exon2) in five cases, (**D**) ROS1 (exon32); SDC4 (exon4) in one case, (**E**) ROS1 (exon34) ;SLC34A2 (exon4) in two cases, (**F**) ROS1 (exon34); SLC34A2 (exon14 del) in three cases, (**G**) ROS1 (exon34); CD74 (exon6) in 17 cases, (**H**) ROS1 (exon34); EZR (exon10) in 13 cases, (**I**) ROS1 (exon35); GOPC (exon8) in one case, and (**J**) ROS1 (exon32) ;SLC34A2 (exon4) in four cases.

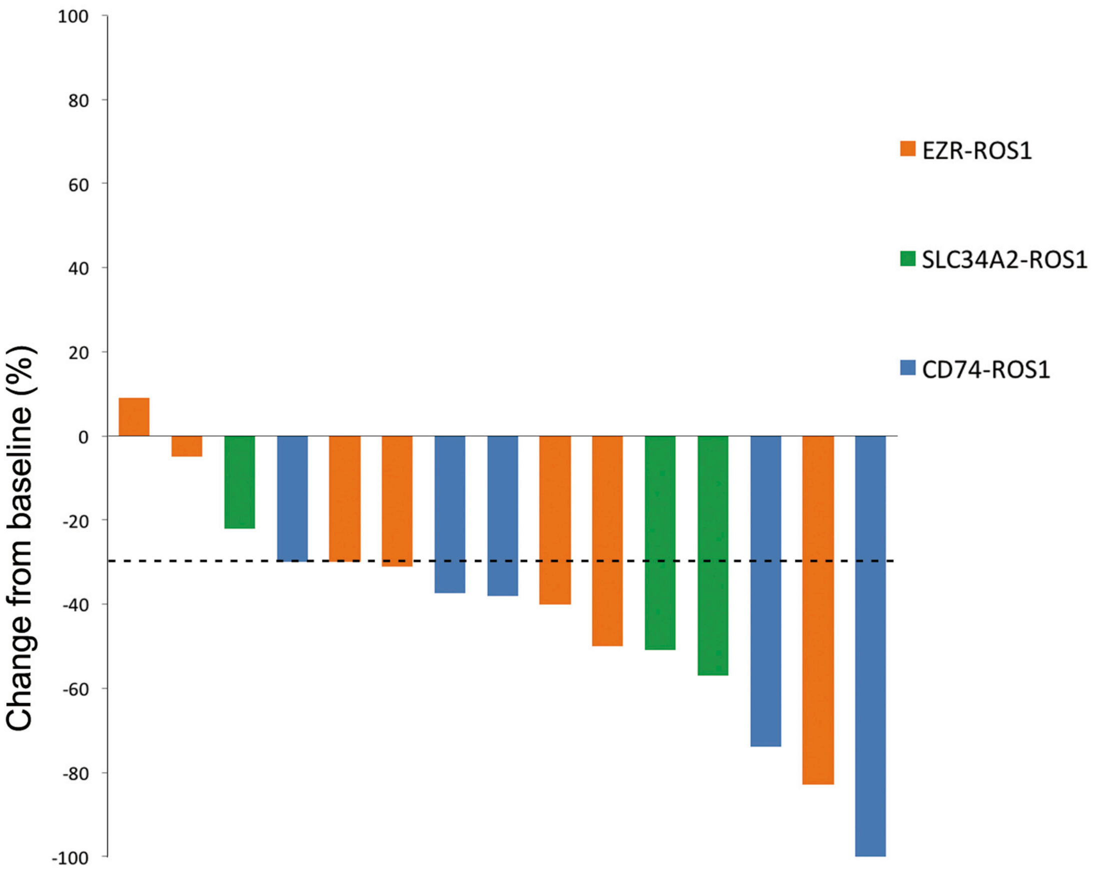

Supplementary Figure S2: Waterfall plot of best percent change from baseline of target lesions for 15 *ROS1* fusion-positive patients with *EZR-ROS1, SLC34A2-ROS1, CD74-ROS1* fusion patterns, respectively.
